# Evaluation of various RNA-seq approaches for identification of gene outrons in the flatworm Opisthorchis felineus

**DOI:** 10.18699/VJ20.688

**Published:** 2020-12

**Authors:** N.I. Ershov, D.E. Maslov, N.P. Bondar

**Affiliations:** Institute of Cytology and Genetics of Siberian Branch of the Russian Academy of Sciences, Novosibirsk, Russia; Novosibirsk State University, Novosibirsk, Russia; Institute of Cytology and Genetics of Siberian Branch of the Russian Academy of Sciences, Novosibirsk, Russia Novosibirsk State University, Novosibirsk, Russia

**Keywords:** opisthorchiasis, spliced leader trans-splicing, outron, start of transcription, transcriptome, ribosomal RNA, описторхоз, сплайс-лидер зависимый транс-сплайсинг, аутрон, старт транскрипции, транскриптом, рибосомальная фракция РНK

## Abstract

The parasitic flatworm Opisthorchis felineus is one of the causative agents of opisthorchiasis in humans. Recently, we assembled the O. felineus genome, but the correct genome annotation by means of standard methods was hampered by the presence of spliced leader trans-splicing (SLTS). As a result of SLTS, the original 5’-end (outron) of the transcripts is replaced by a short spliced leader sequence donated from a specialized SL RNA. SLTS is involved in the RNA processing of more than half of O. felineus genes, making it hard to determine the structure of outrons and bona fide transcription start sites of the corresponding genes and operons, being based solely on mRNA-seq data. In the current study, we tested various experimental approaches for identifying the sequences of outrons in O. felineus using massive parallel sequencing. Two of them were developed by us for targeted sequencing of already processed branched outrons. One was based on sequence-specific reverse transcription from the SL intron toward the 5’-end of the Y-branched outron. The other used outron hybridization with an immobilized single-stranded DNA probe complementary to the SL intron. Additionally, two approaches to the sequencing of rRNA-depleted total RNA were used, allowing the identification of a wider range of transcripts compared to mRNAseq. One is based on the enzymatic elimination of overrepresented cDNAs, the other utilizes exonucleolytic degradation of uncapped RNA by Terminator enzyme. By using the outron-targeting methods, we were not able to obtain the enrichment of RNA preparations by processed outrons, which is most likely indicative of a rapid turnover of these trans-splicing intermediate products. Of the two rRNA depletion methods, a method based on the enzymatic normalization of cDNA (Zymo-Seq RiboFree) showed high efficiency. Compared to mRNA-seq, it provides an approximately twofold increase in the fraction of reads originating from outrons and introns. The results suggest that unprocessed nascent transcripts are the main source of outron sequences in the RNA pool of O. felineus.

## Abstract (ru)

Opisthorchis felineus – представитель паразитических плоских червей, один из возбудителей описторхоза человека. Недавно нами была проведена сборка генома O. felineus, однако корректная аннотация генов в этом геноме стандартными методами оказалась затруднена наличием сплайс-лидер зависимого транс-сплайсинга (SLTS). В результате SLTS исходный 5’-конец (аутрон) транскриптов заменяется короткой сплайс-лидерной последовательностью, донором которой выступает специализированная молекула SL РНК. SLTS вовлечен в процессинг РНК более половины всех генов O. felineus, из-за чего становится невозможным установить последовательности аутронов и реальные старты транскрипции соответствующих генов и оперонов, опираясь только на данные mRNA-seq. В настоящей работе мы провели апробацию различных экспериментальных подходов для идентификации последовательностей аутронов у O. felineus с помощью массового параллельного секвенирования. Два подхода были спланированы нами для прицельного секвенирования процессированных разветвленных аутронов. Первый заключался в сиквенс-специфичной обратной транскрипции с SL-интрона в направлении 5’-конца аутрона. Во втором использовалась гибридизация аутронов с иммобилизованным одноцепочечным ДНК-зондом, комплементарным SL-интрону. Также были использованы два подхода к секвенированию тотальной РНК, обедненной по рРНК, позволяющих идентифицировать более широкий спектр транскриптов, чем mRNA-seq. Один из них основан на ферментативной элиминации перепредставленных кДНК, другой – на ферментативной деградации некэпированных РНК экзонуклеазой Terminator. С помощью селективных методов нам не удалось получить обогащения препаратов РНК по процессированным аутронам, что, наиболее вероятно, связано с коротким временем жизни этих промежуточных продуктов транс-сплайсинга. Из двух методов обеднения по рРНК высокую эффективность показал метод, основанный на ферментативной нормализации кДНК (Zymo-Seq RiboFree). Он позволил примерно вдвое увеличить долю прочтений, соответствующих аутронам и интронам, по сравнению с mRNA-seq. Полученные результаты предполагают, что основным ресурсом последовательностей аутронов в пуле РНК O. felineus служат новосинтезированные непроцессированные транскрипты.

## Introduction

Opisthorchis felineus is a representative of parasitic flatworms (Trematoda: Opisthorchiidae), which has a complex life cycle with two intermediate hosts and the mammalian definitive host, including humans (Beer, 2005). Opisthorchiasis caused by parasitism of this fluke in the bile ducts of the human liver has a chronic course and leads to a number of serious concomitant disorders of the hepatobiliary system, including cholangitis, cholecystitis, pancreatitis, and is also a risk factor for the development of cholangiocarcinoma (Sripa et al., 2007; Pakharukova, Mordvinov, 2016; Pakharukova et al., 2019). According to WHO study, more than 1 million people estimated to be infected with O. felineus, with the largest focus of opisthorchiasis located in the Ob-Irtysh basin, Russia, with the incidence in some regions of the Tomsk and Tyumen regions up to 60 % (FAO/WHO, 2014; Fedorova et al., 2018)

**Fig. 1.**
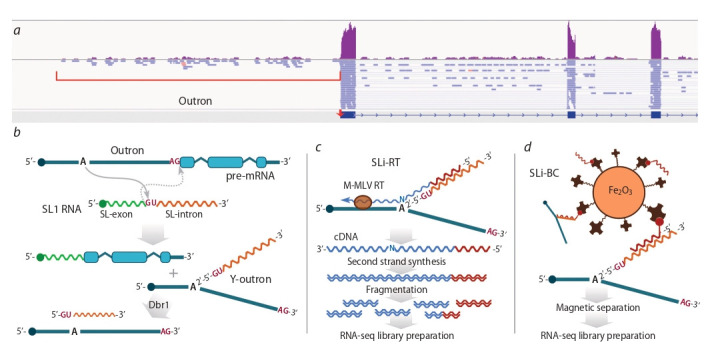
The presence of outronic sequences in the polyA-mRNA-seq data of O. felineus and the graphical overview of the proposed RNA-seq methods for targeted identification of Y-outrons. а – an example of read coverage of an outronic region of a trans-spliced gene (Vps39l) in mRNA-seq data of O. felineus adult worm. The arrow indicates the confirmed trans-splicing site; b – a schematic overview of trans-splicing of pre-RNA with the formation of an Y-branched outron containing an SL-intron arm, and its subsequent degradation with the participation of Dbr1; c – scheme of reverse transcription primed from the SL-intron within the Y-outron, used in the SLi-RT approach; M-MLV RT, M-MLV [H-] reverse transcriptase; d – scheme of hybridization of a single-stranded DNA probe modified with 5’-biotin-TEG, with an SL-intron within the Y-outron, and subsequent purification on magnetic beads coated with streptavidin.

One of the necessary steps to study the biology of this flatworm, and the corresponding development of molecular genetic approaches to the diagnostics and pharmacotherapy of opisthorchiasis, was to obtain a reference genome assembly and its annotation (Ershov et al., 2019). However, the existing gene annotation based on polyA-mRNA-seq data, which is commonly used for this task, has significant drawbacks due to one of the features of RNA processing in flatworms – spliced leader trans-splicing (SLTS). During SLTS, the exons of two independent transcripts are fused: a specialized short capped spliced leader RNA (SL RNA) carrying a 5′-splice site, and one or the other pre-RNA with a corresponding 3′-site (see Fig. 1, b). At the same time, the original 5′-region of the pre-RNA, together with the SL RNA intron attached to its branchpoint through the 2′–5′-phosphodiester bond, is excised (Murphy et al., 1986; Sutton, Boothroyd, 1988). It is assumed that this Y-branched product (Y-outron) undergoes rapid debranching and degradation (Sutton, Boothroyd, 1988; Lasda, Blumental, 2011). Thus, in the polyA-mRNA-seq data, the sequences of excised outrons are practically absent, and with them, localization of the actual transcriptional start sites and promoters of the corresponding genes, as well as information on the operonic organization of gene groups, becomes inaccessible. It should be noted that due to SLTS, a large group of experimental methods for identifying transcription starts based on selective sequencing of the capped 5′-ends of RNAs is also ineffective. 

According to our estimates, products of more than half of all genes in the O. felineus genome undergo trans-splicing (Ershov et al., 2019). Thus, the correct annotation of such genes requires alternative high-throughput sequencing methods for large-scale identification of outrons. Most often, to describe gene outrons, researchers combine information on transcription initiation sites obtained by such methods as GRO-seq, GRO-cap, ChIP-seq to RNA polymerase II, with data on SLTS sites detected by mRNA-seq (Chen et al., 2013; Kruesi et al., 2013). In this way, the region of the genome from the start of transcription to the 3′-site of the SLTS is accepted as an outron. It should be noted that most of the mentioned methods require a large amount of starting biological material and, in the case of a small size of individuals, is suitable mainly for organisms cultured in the laboratory. There are few known examples when it was possible to detect outrons directly by RNA sequencing methods. Thus, in one of the studies on C. elegans, growth of individuals at low temperature led to the accumulation of unprocessed transcripts, which made it possible to detect the 5′-ends of outrons by SAGE of the nuclear fraction of RNA (Saito et al., 2013).

On non-model organisms, including trematodes, RNA-seq “SLTrapping” methods have been successfully used for direct massive detection of trans-spliced mRNAs, based on selection for the universal 5′-sequence of processed transcripts, the SL exon (Nilsson et al., 2010; Boroni et al., 2018). Since all Y-outrons similarly contain the universal 3′-sequence of the SL intron, it seems attractive to use this property for selective identification of outrons and symmetric confirmation of SLTS sites. In addition, direct identification of Y-outrons would serve as direct evidence of the generally accepted mechanism of SLTS on a large sample of genes.

In the current study, to identify O. felineus outrons, we evaluated two candidate approaches based on targeted enrichment for Y-outrons, in one case, by sequence-specific reverse transcription primed from the SL intron towards the 5′-end of the outron, in the other – by hybridization of Y-outrons with an immobilized single-stranded DNA probe complementary to the SL-intron. In addition, two approaches to sequencing of rRNA-depleted total RNA that do not use hybridization probes were used as an alternative: one that use rRNA cleavage by Terminator exonuclease, and the commercial Zymo-Seq RiboFree kit based on enzymatic normalization of cDNA. The proportion of sequences containing outrons in these libraries should certainly be higher than in standard polyA-mRNA-seq libraries.

## Materials and methods

Biomaterial. Adult O. felineus worms were isolated from the bile ducts of golden hamsters Mesocricetus auratus after 3–4 months after infection with metacercariae obtained from the tissues of naturally infected fish (Leuciscus idus) from the Ob river. The worms were washed in saline buffer and used immediately or frozen and stored at –80 °C.

RNA isolation. RNA was isolated from fresh or frozen O. felineus samples using PureZOL (BioRad, USA) according to the manufacturer’s protocol. Precipitation of RNA was carried out by adding an equal volume of isopropanol and 5 μg of linear polyacrylamide (LPA) as a co-precipitant; the resulting solution was left overnight at –20 °C. The precipitate was dissolved in bidistilled water, and additional purification of samples was carried out using an Aurum total RNA mini kit (BioRad, USA), including a stage of treatment with DNase I. The quality and amount of isolated RNA was assessed using a NanoDrop 2000 spectrophotometer

Terminator exonucleolytic cleavage. Total RNA was treated with Terminator enzyme (5′-Phosphate-Dependent Exonuclease, Epicentre, USA) according to the manufacturer’s protocol. The reaction mixture contained 500 ng of total RNA, 1 μl of RiboLock (ThermoFisher Scientific, USA), 2 μl of 10× buffer A, 1 U of Terminator exonuclease. The reaction volume was brought up to 20 μl with bidistilled water. The sample was incubated for 60 min at 30 °C, the reaction was stopped by the addition of 1 μl of 100 mM EDTA. The products of the reaction were purified using Agencourt RNAClean XP beads (Beckman Coulter, USA). The decrease in the amount of ribosomal RNA was checked on an Agilent 2100 Bioanalyzer using an RNA 6000 Pico chip.

Isolation of RNA fraction enriched in Y-outrons (SLiBC). A biotinylated ssDNA probe ([biotin-TEG]-5′-GGC TAGCCAAATAATTCATCCGACCATAGGCCGGAGTC GATTCTT-3′) was immobilized on magnetic beads (Dynabeads M-280 Streptavidin, Invitrogen, USA) in accordance with the manufacturer’s protocol, monitoring the approximate correspondence of the amount of bound probe to the declared binding capacity (~200 pmol/mg). 1 μg of RNA in a buffer for hybridization (1 M NaCl, 5 mM Tris-HCl (pH 7.5), 0.5 mM EDTA, 0.1 % Tween-20) was added to the magnetic beads covered with a DNAprobe and incubated with constant stirring according to the following protocol: 75 °C for 2 minutes, then the temperature was lowered by 1 °C/min to 55 °C and fixed for 15 min at 55 °C. The particles were washed three times with warm (50 °C) buffer. 10 μl of water was added to the particles and heated to 94 °C for 5 min. The RNA-containing solution was collected in a clean tube and the reverse transcription reaction was carried out immediately. For the reverse transcription reaction, 200 U RevertAid Reverse Transcriptase (ThermoFisher Scientific, USA) and random hexamers were used. The reaction was carried out under the following conditions: 5 min at 25 °C, 60 min at 42 °C, and 5 min at 70 °C. The resulting cDNA was used to prepare the library

Reverse transcription reaction with sequence-specific primers (SLi-RT). The thermostable Maxima H Minus Reverse Transcriptase (ThermoFisher Scientific, USA) was used for the reverse transcription reaction. The reaction mixture containing 300 ng RNA, 2 pmol of a specific primer to the SL-RNA intron (SLi_r1, 5′-AGGCCGGAGTCGATTCTT-3′), 1 μl 10 mM dNTP, 4 μl 5 M betaine, was incubated for 2 min at 75 °C, then the temperature was lowered at a rate of 2 °C/min to 55 °C; 20 U RiboLock RNase Inhibitor and 100 U Maxima H Minus Reverse Transcriptase were added and the mixture incubated for 30 min at 55 °C and for 5 min at 85 °C to inactivate the reaction. The resulting cDNA was used to prepare the library.

Evaluation of Y-outron enrichment of SLi-RT and SLi-BC cDNA samples using real-time PCR (RT-PCR). To assess the enrichment for Y-outrons during the elaboration of the SLi-BC and SLi-RT protocols, RT-PCR with primers to the outron (MMCE_ou_f: 5′-CCTGGCGACACACATCTG AA-3′, MMCE_ou_r: 5′-ACATGGACATGGCTGAAGCA-3′) and exons (MMCE_ex_f: 5′-TGCAACCTCTCTTGTGTT CCT-3′, MMCE_ex_r: 5′-CCACCTGGACACCGAATG TAT-3′) of the mmce gene was carried out (Supplementary Material 1)^1^. Enrichment was calculated by the ΔΔCt method as the change in the difference of outron from exon between control and selected cDNA. The control was cDNA obtained by reverse transcription of total RNA primed from random hexamers. In the case of SLi-RT, the selected cDNA was obtained by reverse transcribing the total RNA from the SLi_r1 primer, and in the case of SLi-BC, by converting the enriched RNA fraction using random hexamers.

 Supplementary Materials 1–3 are available in the online version of the paper: http://vavilov.elpub.ru/jour/manager/files/SupplErshov_engl.pdf


PCR was carried out in a 20 μl reaction mixture containing 0.25 mM dNTP, 2 μL 10X PCR buffer B+EVAGreen, 2.5 mM MgCl2, 10 pmol of each primer, 2 μl cDNA sample, 0.3 U/μl SynTaq DNA polymerase with antibodies inhibiting polymerase activity (Syntol, Russia). The reaction was carried out with preheating to 95 °C for 5 minutes followed by 39 amplification cycles including denaturation at 95 °C for 15 seconds, primer annealing and elongation at 60 °C for 20 seconds. Melting curves were acquired within the temperature range 65 to 95 °C. All reactions were performed in two technical replicates.

Library preparation. To prepare RNA-seq libraries after Terminator treatment, NEBNext Ultra II Directional RNA Library Prep Kit for Illumina (NEB, USA) was used according to the standard protocol. To prepare libraries from cDNA samples obtained by the SLi-BC and SLi-RT protocols, NEBNext Ultra II Directional RNA Library Prep Kit for Illumina was used, starting from the stage of the second strand cDNA synthesis. Fragmentation of the cDNA was performed using the dsDNA Fragmentase enzyme simultaneously with the repair of the DNA ends. Fragments of the required length were selected using Agencourt AMPure XP beads, after which several cycles of library amplification were performed.

rRNA-depleted RNA-seq libraries were prepared using the Zymo-Seq RiboFree Total RNA Library Prep Kit (Zymo Research, USA) according to the standard protocol. 500 ng of total RNA was used in the reaction; depletion after renaturation was carried out for 60 min.

The size and quantity of the resulting libraries was determined using an Agilent 2100 Bioanalyzer (Suppl. Material 2). The resulting libraries were sequenced in paired-end mode (2×250 bp) on the Illumina MiSeq platform (the service was provided by Vector-Best, Russia). Raw sequencing data has been deposited in the open-access repository Zenodo (Ershov, 2020). Previously published mRNA-seq data used in the study are available in the NCBI repository (PRJNA257351).

Computational processing of sequencing data. Sequencing data in the FASTQ format was processed using the Cutadapt software (Martin, 2011) to remove adapter sequences and mapped to the O. felineus reference genome (GenBank ID GCA_004794785.1) using the STAR aligner (Dobin et al., 2013). The annotation of genomic elements was constructed using the corresponding gene annotation and previously obtained genome-wide data on positions of trans-splicing sites (Ershov et al., 2019) using in-house Perl scripts. The assembly of the genomic rRNA repeat was carried out manually using the data of genomic DNA sequencing (Ershov et al., 2019). The boundaries of rRNA genes in the genome were determined using the Rfam database (https://rfam.xfam.org) and the RNAmmer tool (Lagesen et al., 2007). Read counts in the defined genomic intervals were computed using the featureCounts tool from the Subread package (Liao et al., 2019). Statistical processing and graphical data visualization were carried out in the R environment.

## Results and discussion

Description of the approaches

At the moment, we have not been able to find any reference in the literature to experimental methods for the direct massive identification of Y-outrons in the transcriptome. Upon examination of the previously obtained O. felineus mRNA-seq data (Ershov et al., 2019), we found that the 5′-regions upstream of the trans-splicing site (potential outrons) of many genes have the same weak read coverage as their introns (Fig. 1, a), despite the depletion of non-coding and unprocessed transcripts as a result of polyA selection. In total, about 0.15 % of mRNA-seq reads were mapped to outronic regions (5 kb upstream of the site) of trans-spliced transcripts (on average with 0.8-fold coverage). The source of such outronic reads can be: (1) unprocessed nascent pre-RNA, (2) Y-outrons – intermediate Y-branched byproducts of trans-splicing, as well as (3) products of their further debranching, which lack the covalently bound SL-intronic arm (see Fig. 1, b). We assumed that the amount of Y-outrons (2) in the total RNA pool could potentially be sufficient for its isolation by targeted enrichment methods.

The possibility of targeting Y-outrons is due to the presence of a universal sequence – an SL-RNA intronic arm, covalently bound at a branchpoint to outron of pre-RNA (see Fig. 1, b). Using this property, we have developed two different approaches to direct selection of Y-outrons. The first, designated SLi-RT (SL-intron Reverse Transcription), is a targeted reverse transcription from the 2′-arm (SL-intron) towards the varying 5′-end of the outron (see Fig. 1, c). It is known that M-MLV reverse transcriptase lacking RNase H activity, when primed from the 2′-arm, is able to quite frequently bypass the branchpoint with the introduction of single-mismatch errors and successfully reverse the 5′-segment (Bitton et al., 2014; Döring, Hurek, 2017). Among the expected drawbacks of the method are the reduced efficiency of such reverse transcription, the accumulation of products of mispriming, and the underrepresentation of the 5′-regions of outrons due to a frequent accidental termination of the first strand cDNA synthesis. In accordance with the latter, the choice of enzyme and high temperature reaction conditions, including hot start, were optimized for maximum processivity and synthesis specificity.

The second approach, SLi-BC (SL-intron Biotin Capture), is based on hybridization of the 2′-arm of outrons with a biotinylated single-stranded DNA probe homologous to the SL intron, followed by immobilization and purification of the corresponding fraction on magnetic particles coated with streptavidin (see Fig. 1, d ). This approach should be more sensitive to the proportion of the target within the total RNA sample, but it has a better chance of identifying full-length outrons.

Both approaches are aimed at the intact Y-structure of the processed outron and therefore would allow differentiating them from fragments of nascent transcripts that did not undergo trans-splicing. In addition, we also applied two methods for sequencing of the rRNA-depleted total RNA, that allow identification of noncoding transcripts, including introns and outrons. At the same time, since O. felineus is a non-model species, available depletion kits based on hybridization with rRNA probes are not applicable. We therefore tested a protocol for treatment of total RNA with the Terminator exonuclease, which specifically hydrolyzes nucleic acids with a phosphorylated 5′-end (including rRNA), but does not affect capped transcripts. Alternatively, we applied the commercial Zymo-Seq RiboFree protocol, based on DSN-normalization of RNA:DNA hybrids immediately after the synthesis of the first cDNA strand, as a result of which all overrepresented transcripts, including globins and rRNA, are hydrolyzed. Unlike the first two, these methods do not make it possible to distinguish the processed outrons from their precursors – unprocessed and nascent transcripts, and therefore cannot serve as direct evidence of the trans-splicing event.

The sequences of the previously identified intact SL-RNA (Ershov et al., 2019) were used to design the corresponding primers and oligonucleotide probes. Optimization of the enrichment conditions for Y-outrons in the SLi-BC and SLi-RT methods was controlled by RT-PCR with primers to the outron and exons of the mmce gene, the trans-spliced product of which is not polycistronic and is among the most highly expressed transcripts (see Suppl. Material 1). The enrichment was calculated as the difference of ΔCt (outron, exon) between the control and the enriched cDNA samples. In the case of cDNA samples used for the subsequent preparation of the SLi-BC and SLi-RT libraries, there was 4- and 100-fold enrichment in the outron region relative to the mmce exons, respectively (see Suppl. Material 1, B).

RNA-seq libraries were prepared from the RNA or cDNA samples obtained by various methods and then sequenced with low coverage, which gives a reasonable indication of the overall performance for enrichment or depletion of target sequences.

Residual rRNA content in libraries

**Fig. 2.**
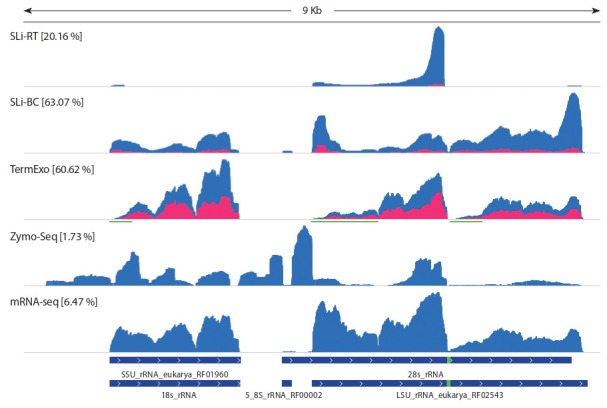
Coverage profiles of genomic rRNA repeat containing 18S, 5.8S, 28Sα, and 28Sβ rDNA. Coverage profiles with antisense and sense cDNA reads are highlighted in blue and red. In the designations of the libraries, the percentage of reads mapped to the rRNA repeat is indicated in square brackets. The dark green lines mark the 5’-regions of rRNA that are most susceptible to hydrolysis by the Terminator exonuclease. The bottom track shows the positions of the rRNA genes predicted by the Rfam and RNAmmer 1.2 services, indicating the hidden break locus of the 28S rRNA (green).

To compare the applied approaches against the standard mRNA-seq method, the obtained sequencing data from five libraries were mapped to a reference genome supplemented with an rRNA repeat sequence. Since the latter repeat had a sufficiently high coverage in all libraries, it turned out to be useful not only for assessing the efficiency of rRNA depletion, but also for assessing the strand-specificity of the resulting libraries (Fig. 2). Of the libraries, only TerminatorExo had a relatively low degree of strand-specificity (68 %). Interestingly, each of the libraries was characterized by its own specific rRNA coverage profile. It is worth mentioning that, for a large number of protostomes, 28S rRNA is additionally processed at the “hidden break” site with the formation of approximately equivalent in size 28Sα and 28Sβ fragments (Ishikawa, 1977). This feature is also observed for O. felineus: a coverage drop in the 28S rRNA (see Fig. 2) and a single rRNA peak in the electropherogram (see Suppl. Material 2)

The observed distribution of the few rRNA reads of the Zymo-Seq library in comparison with mRNA-seq (1.7 and 6.5 % of reads, respectively) reflects extremely effective subtractive depletion in the most overrepresented regions of this repeat.

The TerminatorExo library profile indicates a fairly effective initial enzymatic degradation of the 5′ regions of 18S, 28Sα, and 28Sβ rRNA (see Fig. 2, marked with green lines), which, however, rapidly fades away at certain sites. This is probably due to the sensitivity of the enzyme to the complex tertiary structure of the substrate, or to the frequent hydrolysis of rRNA at these sites with the formation of 5′-OH ends, which protect against further exonucleolytic cleavage by the enzyme. Consequently, the resulting TerminatorExo library contained about 60 % of rRNA reads.

Targeted enrichment methods SLi-BC and SLi-RT also showed high levels of background rRNA (63.1 and 20.2 %). Namely, the SLi-RT library contained mainly the product of off-target reverse transcription from the 3′-region of 28Sα rRNA. Since the 3′-region of 28Sα rRNA can potentially form a stable hairpin (according to the secondary structure predicted by the RNAfold service, Suppl. Material 3), it is likely that such a structure determines the priming of reverse transcription without any participation of the SLi_r1 primer.

Representation of various genomic features in sequenced libraries

**Fig. 3.**
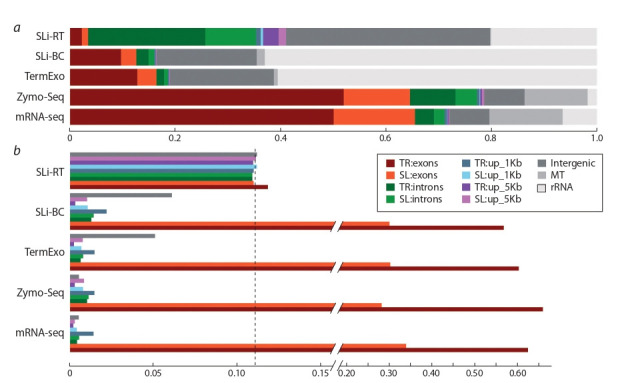
Representation of various genomic elements in the RNA-seq data produced by different approaches. а – fractional distribution of mapped reads across 11 categories of genomic elements; b– chart showing normalized read counts in each of the 9 genomic categories (excluding mtRNA and rRNA). Values are normalized to total category length and sequencing depth (similar to TPM metric). The dashed line indicates the expected level of values under the condition of uniform genome coverage.

For the mapped data, we analyzed the distribution of reads across various mutually exclusive groups of genomic elements in the following priority order: rRNA repeats, mtDNA, exons, introns, promoter or outron regions (1 and 5 Kb upstream of the 5′-end of the predicted gene) and intergenic loci. The calculation was carried out separately for genes undergoing highly efficient trans-splicing (SL) and for all other genes (TR). As can be seen from the results presented in Fig. 3, a, in the TerminatorExo, SLi-BC and SLi-RT libraries, the overwhelming proportion of reads is represented by the uninformative rRNA fraction and the category of intergenic loci. In contrast, in the Zymo-Seq and mRNA-seq libraries, more than 60 % of the reads are mapped to exons of the annotated genes. 

If we exclude the rRNA and mtRNA groups from consideration (see Fig. 3, b), the distribution of read counts across categories of genomic elements, normalized to the total length of the latter, turns out to be quite similar for the mRNA-seq, SLi-BC, TerminatorExo, and Zymo-Seq libraries. The SLi-RT library generally reproduces a random (uniform) distribution of reads across the genome. Obviously, SLi-RT contains mainly noise, which was probably originated from genomic DNA, despite the identical DNase treatment of all RNA samples. It is worth noting that this library contained 227 (0.12 %) read pairs containing the SLi_r1 primer at the beginning of the fragment (next to the adapter sequence). These target sequences were expected to be overrepresented in the SLi-RT library. Although they most likely do not belong to genuine Y-outrons, their presence confirms the efficiency of the primer in the reverse transcription reaction.

**Fig. 4.**
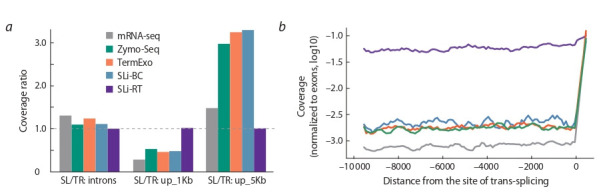
Enrichment of the libraries by outronic sequences а – barplot of the enrichment in introns and outrons of trans-spliced genes (SL) compared to similar regions of non-trans-spliced genes (TR). The coverage is normalized to the total length of the categories; b – profiles of the total normalized coverage of potential outrons in the genome in each of five libraries. The X-axis shows the distance (bp) from the identified trans-splicing site. The color coding corresponds to the legend in section a.

It was assumed that in the case of effective enrichment of libraries by outrons we should observe a significant increase in the coverage of 5′-adjacent regions of trans-spliced genes (SL:up_1Kb and SL:up_5Kb) relative to the promoter regions of ordinary genes (TR:up_1Kb and TR:up_5Kb). This enrichment was indeed observed for the SLi-BC, TerminatorExo and Zymo-Seq libraries, but only in the case of the SL:up_5Kb category (Fig. 4, a). In contrast, these libraries demonstrated depletion in potential outrons in SL:up_1Kb. The depletion is most likely due to the fact that for many “TR” genes their 5′-UTRs are not annotated correctly and therefore a large number of reads originating from mature mRNAs are falsely assigned to the TR:up_1Kb category. More revealing is the comparison with mRNA-seq, in which all three mentioned methods were more enriched in both categories of outronic regions. Since the coefficient of enrichment is approximately the same for the three libraries, it describes just the actual fraction of outrons in the initial total RNA. Consequently, the SLi-BC method did not result in preferential selection of target Y-outron sequences

Thus, of all the tested methods, only Zymo-Seq protocol made it possible to significantly get rid of the uninformative rRNA fraction, while preserving the original fraction of noncoding transcripts, including outrons. At the same time, the coverage of outrons compared to exons in the Zymo-Seq library remains very low (see Fig. 4, b). 

Taken together, these results suggest that sequences corresponding to outrons including unprocessed or nascent transcripts and intact or partially degraded Y-outrons, are represented by a very small fraction in the O. felineus RNA pool. Apparently, intact Y-outrons make up the smallest part of them, and therefore the SLi-BC and SLi-RT libraries targeting the intact Y-outron structure contained mainly unspecific noise. Although the mechanism and rate of degradation of Y-branched trans-splicing products remain unknown, it is assumed that they degrade rapidly (Lasda, Blumenthal, 2011). Thus, in an in vitro experiment on C. elegans, all trans-splicing intermediates were observed, except for the Y-branched outrons (Hannon et al., 1990). 

The main source of noise in the SLi-BC method was the nonspecific sorption of RNA, while in the SLi-RT method, it was off-target reverse transcription of rRNA without the participation of a primer, as well as a minimal admixture of genomic DNA. The detection of the latter only in this library once again indicates that the target RNA template was almost absent in the reverse transcription reaction, namely, outrons with an intact Y-branched structure.

## Conclusion

Thus, of the RNA-seq approaches considered, which theoretically allow identification of the outrons of trans-spliced transcripts, the Zymo-Seq RiboFree approach, which uses enzymatic cDNA normalization, turned out to be the most promising. The ineffectiveness of the targeted SLi-BC and SLi-RT methods is likely due to undetectable amounts of Y-branched outrons in the total RNA pool.

## Conflict of interest

The authors declare no conflict of interest.
